# The Complement System as a Part of Immunometabolic Post-Exercise Response in Adipose and Muscle Tissue

**DOI:** 10.3390/ijms252111608

**Published:** 2024-10-29

**Authors:** Bartosz Wojciuk, Ignacy Frulenko, Andrzej Brodkiewicz, Dagmara Kita, Monica Baluta, Filip Jędrzejczyk, Marta Budkowska, Karolina Turkiewicz, Patrizia Proia, Andrzej Ciechanowicz, Dorota Kostrzewa-Nowak, Robert Nowak

**Affiliations:** 1 Department of Diagnostic Immunology, Chair of Microbiology, Immunology and Laboratory Medicine, Pomeranian Medical University in Szczecin, 72 Powstańców Wlkp. Al., 70-111 Szczecin, Poland; bartosz.wojciuk@pum.edu.pl; 2 Pomeranian Medical University in Szczecin, 1 Rybacka St., 70-204 Szczecin, Poland; frulenko.ignacy@gmail.com; 3 Department of Pathology, Pomeranian Medical University in Szczecin, 1 Unii Lubelskiej St., 71-242 Szczecin, Poland; 4 Department of Pediatrics, Pediatric Nephrology, Dialysis and Acute Intoxications, Pomeranian Medical University, 4 Mączna St., 70-204 Szczecin, Poland; andrzej.brodkiewicz@pum.edu.pl (A.B.); d.kita@szpital-zdroje.pl (D.K.); m.baluta@szpital-zdroje.pl (M.B.); f.jedrzejczyk@szpital-zdroje.pl (F.J.); 5 Department of Medical Analytics, Pomeranian Medical University of Szczecin, 72 Powstańców Wlkp. Al., 70-111 Szczecin, Poland; marta.budkowska@pum.edu.pl; 6 Department of Laboratory Diagnostics, University Clinical Hospital No. 2, Pomeranian Medical University in Szczecin, 72 Powstańców Wlkp. Al., 70-111 Szczecin, Poland; karolina.turkiewicz@usk2.szczecin.pl; 7 Department of Clinical and Molecular Biochemistry, Pomeranian Medical University in Szczecin, 72 Powstańców Wlkp. Al., 70-111 Szczecin, Poland; andrzej.ciechanowicz@pum.edu.pl; 8 Sport and Exercise Sciences Research Unit, Department of Psychology, Educational Science and Human Movement, University of Palermo, 90144 Palermo, Italy; patrizia.proia@unipa.it; 9 Department of Biopharmaceutics and Pharmacodynamics, Faculty of Pharmacy, Medical University of Gdańsk, 107 Hallera St., 80-416 Gdańsk, Poland; 10 Institute of Physical Culture Sciences, University of Szczecin, 17C Narutowicza St., 70-240 Szczecin, Poland

**Keywords:** innate immunity, cleaved complement protein, post-effort immunity, adiponectin, muscle tissue

## Abstract

The precise molecular processes underlying the complement’s activation, which follows exposure to physical stress still remain to be fully elucidated. However, some possible mechanisms could play a role in initiating changes in the complement’s activity, which are observed post-exposure to physical stress stimuli. These are mainly based on metabolic shifts that occur in the microenvironment of muscle tissue while performing its function with increased intensity, as well as the adipose tissue’s role in sterile inflammation and adipokine secretion. This review aims to discuss the current opinions on the possible link between the complement activation and diet, age, sex, and health disorders with a particular emphasis on endocrinopathies and, furthermore, the type of physical activity and overall physical fitness. It has been indicated that regular physical activity incorporated into therapeutic strategies potentially improves the management of particular diseases, such as, e.g., autoimmune conditions. Moreover, it represents a favorable influence on immunoaging processes. A better understanding of the complement system’s interaction with physical activity will support established clinical therapies targeting complement components.

## 1. Introduction—The Complement Proteins’ Origin, Activation, and Regulation

The complement system constitutes more than 30 plasma and membrane proteins, which play an essential role in maintaining the human organisms’ reactivity to various stimuli, mostly bacterial pathogens, but also physical activity and oxidative stress [1,2,3,4]. Although it represents a group of phylogenetically old immune system components, it fulfills more complex functions than just sustaining innate immunity itself [5,6]. It has also been indicated that physical activity stimulates inflammation [7,8,9,10,11,12]. Inflammatory response, in turn, catalyzes muscle repair and regeneration [13,14,15]. Additional factors which influence the inflammatory response are emotional and mental aspects, related to systemic fatigue [16,17,18,19]. Different sorts of stress (physical, cellular, or psychological), acting separately or in combination, initiate the release of endogenous factors, known as danger- or damage-associated molecular patterns (DAMPs). These further induce sterile inflammation—an inflammatory process triggered without of exogenous toxic agents, such as pathogens [20,21,22,23]. Within the sterile inflammation theory [24,25], this perspective aids in comprehending the impact of high-intensity physical activity on immune system modulation. Post-intense-stimulating modulation occurs at both the extracellular and molecular levels, encompassing not only intracellular signaling but also epigenetic modifications. This modulation is a primary contributor to persistent individual variability [26,27]. Intracellular signaling processes frequently culminate in the expression of additional signaling proteins, of which autocrine or paracrine functions initiate a cascade of cellular responses within a given tissue. This review focuses on the interplay between physical activity, adipose tissue status, and the complement system. Furthermore, molecular mechanisms which activate the complement can take place consequently to exposure to different bouts of exercise. This is mostly due to their immunometabolic functions and their contribution to inflammatory processes. The authors offer an explanation on why measuring the levels of complement components in their activated or deactivated forms post-physical exercise has yielded inconsistent results.

The number of 45 genes, distributed throughout multiple chromosomal loci within the human genome, has been identified to encode the building blocks of the complement system [28]. Most of the complement proteins are present in the plasma in an inactive form, produced by the liver [29,30]. However, populations of mast cells, macrophages (Mφs), dendritic cells (DCs), polymorphonuclear leukocytes, monocytes (MCs), NK cells, B and T cells, and smooth muscle cells (SMCs) have also been recognized to produce both plasma- and membrane-derived components [31,32,33,34]. Apart from these, several cell populations, traditionally not identified as immunocompetent, have also been indicated to express genes involved in encoding components of the complement cascade. These are mainly of mesenchymal origin and include fibromyocytes, myofibroblasts, lipofibroblasts, and adventitial fibroblasts [35]. Recent findings also point to kidney epithelial cells and retinal cells expressing complement genes, in these cases, under TGF-β1 and IL-1α stimulation [36,37,38,39,40]. However, what must be noted is that the gene expression, indicated as the presence of mRNA in the cytoplasm does not directly correlate with a relevant protein expression [41]. Regardless of the exact level the complement expression in peripheral extra-hepatic tissues (as reported in The Human Protein Atlas—accessed on 19 January 2024—https://www.proteinatlas.org/search/complement), the abovementioned findings point to the complement’s significance in a variety of physiological and pathological processes.

**Figure 1:**
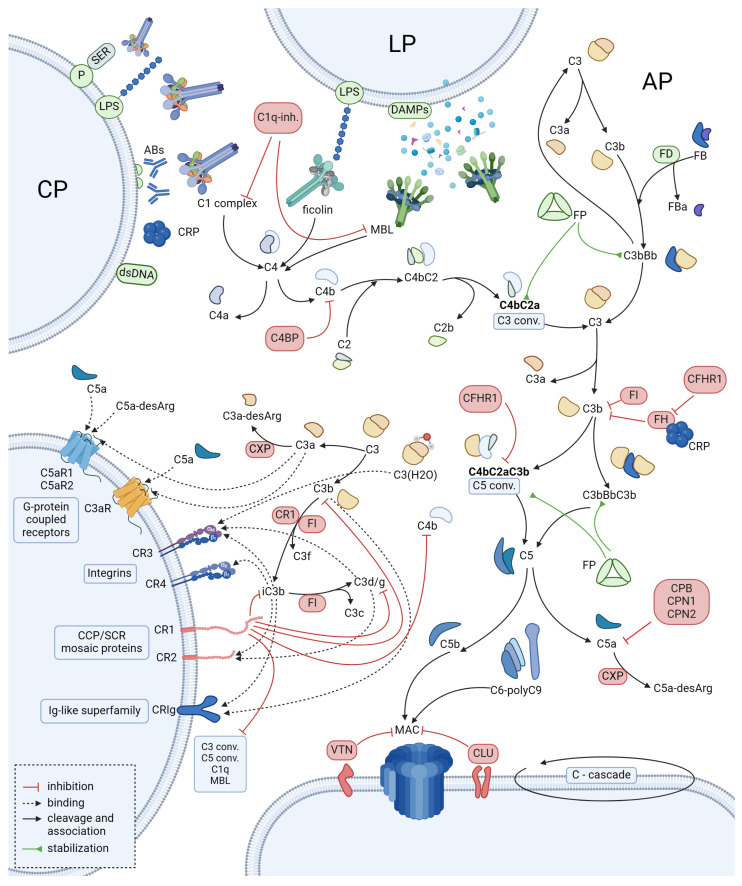
Visual representation of the complement system’s activation, including fluid-phase-based and membrane-bound regulatory components (Created in BioRender.com). The complement cascade is a process which takes place both in the extracellular milieu and in direct contact with cell membranes, much like the coagulation cascade. The upper portion of this Figure depicts processes and molecules that initiate the cascade. Complement activation regulators that have an inhibitory effect have been marked with red; factors that accelerate the cascade or serve as necessary cofactors are indicated by green. It must be emphasized that the AP is a self-perpetuating, constantly active process, albeit with variable intensity, which depends on multiple factors, discussed in more detail in the following passages. On the left side of this Figure, there are membrane bound receptors that either serve as signal transducers (e.g., GPCRs) or as complement activation inhibitors (CR1—along with CR2 is a member of the complement control protein (CCP)/short consensus repeat (SCR) domain modular single-pass transmembrane receptors) keeping its excessive activation at bay and thus preventing cellular injury, which could possibly be caused by AP activation. It has been depicted by again drawing C3 cleavage closely to this Figure section. C3, following its spontaneous cleavage, generates additional fragments, which are subject to proteolysis by carboxypeptidases (CXP); this is how C3-desArg arises. C3b is cleaved by the CR1 receptor and FI to C3f and iC3b. iC3b is an opsonin, which is bound and inhibited by the membrane-bound form of CR1. Abbreviations used in this Figure: Classical pathway (CP), lectin pathway (LP), alternative pathway (AP), phosphatidylserine (P-SER), antibodies (ABs), damage/danger-associated molecular patterns (DAMPs), lipopolysaccharide (LPS), double-stranded DNA (dsDNA), C-reactive protein (CRP), C1q inhibitor (C1q-inh.), factor D (FD), factor B (FB), factor P (FP), C4 binding protein (C4BP), carboxypeptidase N (CXP), complement factor H related protein 1 (CFHR1), factor I (FI), factor H (FH), carboxypeptidase B (CPB), carboxypeptidase N (CPN1/2), C3 convertase (C3-conv.), C5 convertase (C5-conv.).

Three conventional pathways of complement activation—classical, CP; alternative, AP; and lectin, LP—ultimately lead to the formation of the complement’s terminal structure, known as the membrane attack complex (MAC). The membrane attack complex is responsible for cell lysis, which is the major antimicrobial effectory mechanism provided by the complement (Figure 1). Two enzymatic complexes called C3 and C5 convertase represent the critical stages in each pathway.

The classical pathway of complement activation is classified as a crucial component of both the innate and adaptive immune systems [42]. While it primarily relies on antibodies (IgM or IgG) binding to antigens on pathogens—reflecting adaptive immunity—it also activates innate immune responses [43,44]. By enhancing opsonization and promoting inflammation, the classical pathway facilitates pathogen clearance and underscores the interconnectedness of these two branches of the immune response [42]. The lectin complement system pathway is similar to the classical pathway but is independent of immunoglobulins [42]. In turn, activation of the alternative complement system pathway is activated spontaneously by hydrolysis of the internal C3 thioester bond and further triggered by contact with various proteins, lipids, and carbohydrate structures on microorganisms and other foreign surfaces [45,46,47,48].

Several mechanisms are in place to prevent excessive activation of the complement. Specific proteins play a critical role, namely complement inhibitors (CIs). Most of these are expressed either on the surface of host cells or within the extracellular matrix (ECM). Some, such as factor H (FH), factor I (FI), or C4-binding protein, are present in the plasma. Among these, a member of the serpin superfamily C1-inhibitor acts on the classical pathway by inhibiting C1r and C1s and also on the lectin pathway by inhibiting MASP1 (MBL-associated serine protease 1) and MASP2 (MBL-associated serine protease 2) [49]. CIs appear to be particularly important considering that, as mentioned above, the alternative pathway is a constantly self-activating system. CIs, therefore, exert a protective function over healthy cells and keep the self-perpetuating complement system at bay. Furthermore, all three complement pathways are highly linked in terms of regulation. For example, MASP3 of the LP influences the activity of the AP through the cleavage of nascent factor D (FD) into mature FD [50]. C3 convertase formation is inhibited by complement FH, which additionally, through interaction with FI, lyses C3b and consequently inhibits the AP. Other proteins such as vitronectin, clusterin, and pentraxins also exert inhibitory effects towards the complement cascade. These are present both in the plasma and extracellular matrix and inhibit the lytic activity of the complement. Pentraxins are of particular interest, as their action appears contradictory—simultaneously enhancing CP and inhibiting the AP. Furthermore, these are represented by C-reactive protein (CRP), one of the best-recognized clinical inflammatory biomarkers. CRP has an activating influence on the CP, leading to C3b deposition in tissue areas exposed to injury. At the same time, CRP binds FH and potentially routes it to those areas, limiting the AP activation [51,52,53]. The modes of action of the mentioned and additional regulators of complement activity (RCAs) have been summarized in Table 1.

## 2. Functions of Individual Components and Their Cellular Receptor Interactions

Complement receptors are widely distributed across the immune system. These represent a heterogenous group and include glycoproteins (e.g., CR1, CR2), integrins (e.g., CR3, CR4), immunoglobulin-like molecules (e.g., CRIg, an IgV family of Ig-like domains), and G-protein coupled receptors (e.g., C3aR, C5aR1, and C5aR2) [33,66]. Complement components interact with a number of immunocompetent cells [67]. The detailed functional characteristics of particular complement components and associated receptors involved in signal transduction are described in Table 2 and Table 3, respectively.

The complement’s role in antimicrobial immunity is highlighted by disease entities arising due to complement deficiencies other than C1 and C2. Low levels of terminal fragments (C5–C9) and positive regulators of earlier fragments (FP or properdin) are associated with recurrent and severe *Neisseria meningitidis* infections [72,73]. Furthermore, complement proteins control the deposition of immune complexes (ICs) in tissues. The deficiency of this function is seen in autoimmune diseases, with a dominant component of type III hypersensitivity, as described by Gell and Coombs in 1963 [74,75]. Decreased ICs clearance can arise due to genetic polymorphisms and diverse expression of complement receptors on Mφs and erythrocytes, as seen with CR1 in systemic lupus erythematosus (SLE) [28,75]. Furthermore, it can result from hereditary C1 and C2 deficiencies, clinically revealed as SLE-like syndrome or discoid lupus erythematosus (specifically C2 deficiency) [76,77,78,79,80,81].

## 3. Possible Molecular Mechanisms of Complement Activation and Its Effects on Muscle and Adipose Tissues in Active and Sedentary States

In a synoptical perspective, whether the source of inflammation or disease is exo- or endogenous, the complement is omnipresent and one of the most far-reaching mecha-nisms which initiate and regulate immune response. Complement proteins and their respective receptors are involved in the pathogenesis and progression of other multiple inflammatory and systemic diseases, such as frontotemporal dementia, multiple sclerosis, sepsis-induced multiple organ failure, asthma, cystic fibrosis, pulmonary interstitial dis-eases, and pulmonary cancer. They can present with increased plasma, cerebrospinal fluid, and tissue levels of complement proteins and increased receptor expression, both on infiltrating and tissue-specific cells. The complement’s influence on disease course is thus significantly potentiated [76,77,78,79,80,81]. Such imbalances contribute to the enhancement of local tissue destruction. Furthermore, this causes morphological and functional organ damage, which is associated with poor prognosis and is therefore viewed as a therapeutic target [28,32,40,75,77,79,80]. As indicated above, the knowledge on complement origin and its functions beyond immunity sensu stricto has been expanding.

The precise molecular processes underlying the complement’s activation following exposure to physical stress still remain to be fully elucidated. However, some plausible mechanisms could play a role in initiating changes to the complement’s activity and are observed post-exposure to physical stress stimuli. These are mainly based on metabolic shifts that occur within the microenvironment of skeletal muscle and the adipose tissue’s role in sterile inflammation and adipokine secretion.

### 3.1. Skeletal Muscle Strain, Injury, and the Complement’s Regulatory Role in Regeneration

Complement activity following increased muscle use seems to be associated with metabolites present within the extra- and intracellular milieu. One major example of those metabolites is lactate. This organic ion is accumulated in the cytoplasm of muscle cells when they start to utilize stored glycogen due to repetitive contractions. The glucose released during glycogenolysis is then routed to the glycolytic pathway. During intense physical exercise, certain amounts of pyruvate produced in glycolysis cannot be oxidized in the Krebs cycle. Instead, they are used to oxidize the reduced form of nicotinamide adenine dinucleotide (NADH) by lactate dehydrogenase and are reduced to lactate. After that, to some extent, it is extruded out of the cell by lactate–proton transporter proteins. The conditions that arise with intense muscle use provoke the increase in lactate and H^+^ ions. They determine the pH of both the inner milieu of the myocyte and its surroundings [82].

In vitro models have shown that the complement can be activated by both a decrease in pH caused by, e.g., hypercapnia in respiratory acidosis, and increased amounts of lactate in a dose-dependent manner, as seen in lactic acidosis. It is debatable whether it is lactate that activates the complement or the H^+^ ions released during lactic acid dissociation or possibly other metabolic processes [82,83,84,85]. It is particularly difficult to establish whether there is a direct cause-and-effect relationship between increased levels of lactate and complement activation in vivo, because intervention studies with a physical exercise component have not confirmed these outcomes. Navarro-Sanz et al. have, for example, established no correlation between plasma lactate and C4 levels [86]. It is also possible that this interaction between lactate and the complement appears locally and, as of today, remains imperceptible.

Micro-injuries sustained by muscle fibers exposed to strenuous use result in the release of DAMPs, which can initiate the activation of the complement cascade. DAMPs represent a diverse group of molecules, which are released when a cell has either sustained a specific form of sub-lethal injury, has primed itself to undergo apoptosis, is undergoing apoptosis, or when no longer capable of dying through apoptosis, becomes necrotic [87,88]. The exact source and nature of DAMPs, which are possibly released when a muscle cell sustains a sub-lethal injury, have not been determined. Subjection of cultured myocytes to mechanical loads has been shown to induce wound-like cell membrane lesions, allowing for cytosolic proteins to escape into the extracellular milieu [89].

An exemplary molecule which has been shown to activate the complement and is particularly relevant regarding skeletal muscle is a structural protein—desmin. Although not directly associated with DAMPs, it is a cytoskeletal protein specifically expressed in muscle cells, that may be functionally affected during strenuous muscle use. This was demonstrated by the loss or the decrease in immunohistochemical staining against desmin in muscle tissue obtained from rabbits and subjected to forced ankle plantar–flexion by electrical stimulation of the peroneal nerve [13,90,91,92].

While muscle damage causes the complement’s activation, its components can in-duce muscle damage themselves [93,94]. Animal models have demonstrated the complement’s involvement in early stages of increased muscle use and its role in the modulation of the immune system’s response to muscle damage. Using the technique of Morey-Holton and Wronski to study modified muscle use by un- and reloading hindlimbs of Wistar rats, Frenette at al. found that both the AP and CP are involved in modulating chemotaxis of neutrophils and ED1+ Mφs infiltrating the muscle tissue. Furthermore, inhibition of the complement activation by the soluble form of complement receptor-1 (sCR1) (see Table 3), which binds C3b and C4b, has caused a significant reduction in muscle inflammation, necrosis, and edema. A similar effect was observed regarding the concentration of ED1+ cells per mm^3^ of tissue and their infiltration of muscle fibers [95]. The initiated cascade might, therefore, have a different function than merely inducing inflammation. It seems to play a somewhat pronounced role in regulating the step following the inflammation: the resolution.

Zhang et al. have demonstrated that myocytes of wild-type C57BL/6J mice exhibit an increase in expression of genes involved in production of specific complement fragments (*Cfb*, *Cfd*, *Cfp*, *C1qa*, *C1qb*, *C1qc*) and genes encoding the complement receptors (*C3ar1* and *C5ar1*) when exposed to chemical injury caused by cardiotoxin injection [96]. The same study documented the deposition of activated C3 fragments (C3b/iC3b) in the injured myofibers and that it is the AP which plays the most critical role in myofibers injury and repair. When assessing complement fragments, complement receptors, and cells involved in the regeneration processes of damaged muscles, the researchers observed that the interaction between C3a and C3aR, rather than C5a–C5aR, regulates the initiation of recruitment of circulating MCs into the injured muscles, where they contribute to myofiber regeneration. They suggest that this interaction is essential at the early stages of this process [96]. The increase in specific cleaved complement components observed in human serum following physical exercise has been hypothesized to aid the Mφs in the targeting and clearance of debris derived from damaged muscle tissue [97]. It is conceivable that in humans, in addition to the functions mentioned above, individual complement fragments prime circulating cells, e.g., MCs, which then regulate muscle regeneration.

Complement fragments usually interact with membrane-bound receptors, their activation is followed by a series of downstream processes, which regulate, e.g., chemotaxis. However, a murine in vitro model with embryonic muscle-resident pre-adipocytes has shown C3 to induce myogenic differentiation by internalization [98]. In contrast, usually it is the Wnt/beta-catenin-signaling pathway that is associated with differentiation towards cells of myogenic phenotype [98,99].

Skeletal muscles are attached to bones by tendons. Their role is to transfer force which arises due to muscle contracture onto the skeleton, allowing a given movement to be exercised. As well as muscles, these can become injured when the physical force, or its duration, overpowers their resistance capabilities. Tendons are composed of dense connective tissue, predominantly comprising water and collagen, which is synthesized by fibroblasts. Attempts to classify those cells by using transcriptomics have revealed their outstanding heterogeneity, both within a given organ and between organs [100].

A recent study has shown that FD might possibly be implicated in modulating fibroblast migration and collagen production during tendon regeneration. When analyzing proteomic profiles obtained from biopsies of Achilles tendons during reparative surgery, Chen et al. found a significant association between FD and patient outcomes during both the inflammatory and proliferative healing stages, albeit with contrasting effects. Notably, heightened FD levels were observed during the inflammatory phase, while diminished expression was noted during the proliferative healing phase in patients with favorable outcomes compared to those with poor outcomes. The researchers confirmed the stimulatory role of FD in fibroblast migration by an in vitro wound model. Furthermore, their findings indicate that lower expression of FD during the proliferation stage results in an increased production of alpha-1 collagen type 1 by fibroblasts, the primary kind of collagen found in tendons [101].

### 3.2. Regulation of Metabolic Processes Within the Adipose Tissue

Human adipose tissue consists of adipocytes and the supporting stroma of connective tissues. As with any other soft tissue, it is supplied with oxygen by a network of blood vessels and innervated by branches of different efferent, afferent, somatic, and autonomic nerves. The most abundant adipocytes present in the human body are classified as white (WA) and two other distinct populations, namely beige (BeA) and brown adipocytes (BA). They differ in their primary functions and metabolic capabilities, which translates to different secretomes and possible modulatory effects on the complement [102,103,104]. BAs, which are observed in increased amounts in newborns, infants, and during very early childhood, are located primarily para-axially along the cervical vertebral column. However, the existence of BAs is not limited to the period of infancy. Their presence has also been confirmed in adult populations [102,105,106]. The peripheral subcutaneous and visceral fat tissue consists of WA. This population is far greater than BAs or BeAs, particularly in adults, as brown fat tissue regresses with age [105,107].

The adipose tissue produces a set of proteins which directly partake in the complement activation cascade or function as fluid-phase-based RCAs. Their role seems to extend beyond immune defense and to be more centered around regulating local metabolic state along with influencing cell differentiation [108]. However, the adipose tissue does not remain neutral in the setting of immune regulation and response to pathogens. WA have been shown to respond with activation of complement-related genes when stimulated with PAMPs such as LPS in vitro. Matsunaga et al. reported an increase in *Cfb* gene expression following LPS stimulation, while *Cfd*, *Cfh,* and *C3* expressions were not influenced. Additionally, after transfection of adipocytes with *Cfb*, a significant increase in cell size and lipid droplet accumulation was observed. Transgenic mice overexpressing *Cfb* exhibited higher amounts of both subcutaneous white adipose tissue and inguinal white adipose tissue. Enhanced *C3* and *Cfd* expressions accompanied those changes, while *Cfh* expression, a negative RCA, was downregulated. The weight and cell size of brown adipose tissue did not differ between transgenic mice and controls. This study showed that FB appears to play a role in regulating the differentiation of preadipocytes to mature adipocytes [109].

Coan et al. found that *Cfb*-knock-out rats had an altered adipose tissue distribution compared to spontaneously hypertensive rats. They observed a statistically significant reduction in relative wet mass of the visceral adipose tissue and an increase in subcutaneous adipose tissue. The total fat mass was similar between the two groups. Disabling *Cfb* was followed by a favorable change in factors directly involved in the pathogenesis of the metabolic syndrome, such as favorable adipose tissue distribution and a fall in cholesterol, triglyceride, and high-molecular weight adiponectin levels. The authors theorize that, in line with their findings, FB could be a potential therapeutic target in treating human metabolic syndrome [110].

Indeed, increased amounts of visceral white adipose tissue are associated with an increased risk for metabolic syndrome [111,112]. A broad secretome analysis of BAs revealed their capability to secrete FH. We have already mentioned its role in inhibiting the complement cascade through interactions with AP C3-convertase, FI, and CRP. Deshmukh et al. suggested that this finding could translate to better anti-inflammatory capabilities of the brown adipose tissue [103]. However, this matter remains in a speculatory realm.

In humans increased expression of *CFH* along with *CFB* in the white adipose tissue and increased concentration of free FH and FB are significantly associated with insulin resistance and other unfavorable metabolic parameters (blood pressure and fasting triglycerides). *CFH* expression was higher in subcutaneous fat tissue and *CFB* in visceral fat tissue. Interestingly, the increased expressions of the mentioned genes were observed not in adipocytes but in stromovascular cells [113].

FD, a positive RCA important for the function of the AP, can promote adipocyte differentiation and lipid accumulation. Its proposed mechanism of mediating those pro-adipogenic effects is by interacting with C3aR and increasing C3a production [114]. Of the two main anatomically classified fat tissues, the visceral fat tissue seems to play the most pronounced role in secretion of different complement proteins. The study on adult obese men undergoing laparoscopic bariatric surgery, which investigated the expression of complement genes, showed that *C2*, *C3*, *C4*, *C7*, and *CFB* had higher expressions in omental than subcutaneous adipose tissue. The relative expression of *C3* in the omental adipose tissue, compared to beta-actin as an internal reference gene, was 10% of that in the liver. Serum C4 levels in both genders correlated with visceral fat area, corresponding to the measured high levels of *C4* transcripts in the omental adipose tissue. Serum C4 also correlated with subcutaneous fat area and BMI in women but not in men. Interestingly, omental adipose tissue showed higher expression of *C7* compared with liver [115].

The binding of C3-desArg to the C5L2 receptor, which belongs to the same class of complement metabotropic receptors as C3aR, C5aR1, and C5aR2, has been shown to increase fat storage and glucose transport [116,117,118,119]. In rats, deleting the gene for this receptor resulted in increased infiltration of the visceral adipose tissue by Mφs and better chemoattractant capabilities of the C5L2−/− adipocyte-conditioned medium. Gauvreau et al. propose that due to the loss of the C5L2-postulated decoy function (through binding of C5a, C5L2 makes it unavailable for interacting with C5aR), binding of the now more bioavailable C5a to C5aR enhances the chemotaxis of the Mφs [120].

C3a and C5a, when bound to their respective receptors expressed by the adipocytes, can promote energy conservation. They perform this function by increasing the uptake of fatty acids and glucose. Furthermore, they have been shown to reduce cellular concentrations of cyclic adenosine monophosphate by acting through their respective metabotropic receptors (C3aR and C5aR) and inducing PGE2 secretion by Mφs. A murine model has shown that inhibiting those receptors in vivo can ameliorate factors implicated in the pathogenesis of the multiple metabolic conditions, including cardiac fibrosis, glucose tolerance, and liver enzyme concentrations [121].

**Figure 2:**
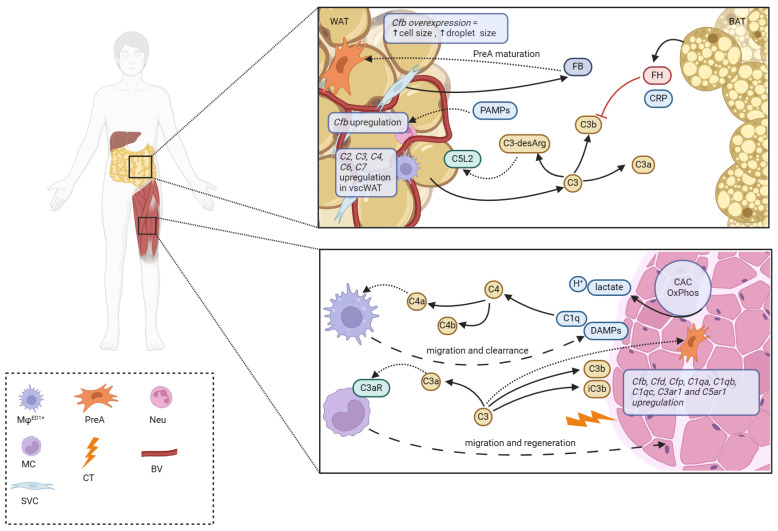
Mechanisms of possible complement involvement in the immunometabolism of adipose and muscle tissue (Created in BioRender.com). Neutrophil (Neu), Monocyte (MC), blood vessels (BV). Finely dashed arrows indicate binding, widely dashed arrows indicate cell migration, line arrows indicate cleavage and association. **Upper Panel**: (1) Stimulation of white adipose tissue (WAT) results in *Cfb* gene overexpression and increases cell and lipid droplet size. (2) Factor B (FB) secreted by stromovascular cells (SVC) induces preadipocyte (PreA) maturation. (3) WAT secretes C3 which then participates in the activation cascade. C3-desArg produced by a carboxypeptidase binds C5L2 and increases fat storage and glucose transport. *C2*, *C3*, *C4*, *C7*, and *Cfb* genes are highly expressed within the visceral WAT (vscWAT). (4) Brown adipose tissue (BAT) produces factor H (FH) which interacts with C reactive protein (CRP) and is a negative regulator of the complement cascade. **Lower Pannel**: (1) Citric acid cycle (CAC) and subsequent oxidative phosphorylation (OxPhos) are sources of hydrogen ions which influence local pH and are capable of complement activation. (2) Damage/danger-associated molecular patterns (DAMPs) are bound by C1q. C1-complex activates the cascade, along which C4 is cleaved and attracts Mφs, which infiltrate the muscle tissue and aid in clearance of DAMPs. (3) Chemical damage by cardiotoxin (CT) induces the deposition of C3b and iC3b on the surface of muscle fibers. C3a, generated by cleavage of C3, binds to C3aR and attracts MCs, which partake in muscle fiber regeneration.

Undoubtedly, physical exercise, accompanied by a proper calorie-deficient dietary regimen, has been proven to be a successful measure to change the human body’s tissue composition and decrease the adipose tissue’s mass. In a sedentary lifestyle characterized by low doses of physical activity, the adipose tissue seems to be prone to dysregulation, of which the complement appears to play an important part. More research is needed to elucidate how physical exercise influences the expression profile of complement-related proteins and how does the activity of the AP, which seems to play the most pronounced role in regulating adipocyte biology, respond to a transition from a sedentary to an active lifestyle in humans on a molecular and systemic level. The possible mechanism of complement involvement in the immunometabolism of adipose and muscle tissue is presented in Figure 2.

## 4. Concluding Remarks

The complement system has been indicated to contribute to processes outside innate immunity. These refer to highly metabolically active elements, such as muscle cells and adipocytes. In the muscles, the complement is strongly considered to contribute to tissue regeneration, the pivotal process following physical activity. In contrast, this activity appears more pleiotropic in the adipose tissue and addresses both local immunity and biochemical status. What seems remarkable is that in both sites, the complement components act indirectly via Mφs. The role of Mφs in tissues regeneration has been previously indicated. These findings correspond with the aforementioned data on complement in muscles and place the complement system as an additional player in regenerative processes. However, this relationship seems more complex in the adipose tissue and involves the complement system, Mφs, and the adipocytes themselves. Despite the established knowledge about dietary- and lifestyle-related influence on adipose tissue status as well as expanding research on macrophages shaping this [122], more data are still needed to understand the precise local interplay between lifestyle-associated molecular patterns (LAMPs), the complement, cellular components, and metabolism.

The immunomodulative effect of physical activity and physical effort is directly related with therapeutic potential of post-effort activation of the complement system. This effect is associated with enhanced regulation of inflammatory response and significant decrease in chronic inflammation associated with numerous diseases, including atherosclerosis, type 2 diabetes, obesity, or rheumatoid arthritis. Additionally it holds promise in cancer immunotherapy. Furthermore, its therapeutic potential is related to the widely discussed involvement of the complement system in tissue regeneration and injury repair processes. This role of the complement system can also lead to decreasing the risks of chronic inflammation. It is also worth emphasizing that the immunomodulative effects of physical activity can serve as tools in preventing the development of many lifestyle-associated diseases, e.g., obesity, type 2 diabetes, or cardiovascular diseases. Properly designed, individualized exercise plans undoubtedly have a significant preventive potential in modulating the activity of the complement system, which may be crucial in the prevention and treatment of many diseases of inflammatory and immunological etiology. Hence, it seems that better understanding the post-effort complement system activation may be an important element in supporting the classical therapeutic methods based on pharmacological therapies.

## Figures and Tables

**Table 1:** The selected regulators of complement system activation and their ligands.

Regulator of Complement Activation	Ligands and Action
Factor H	Negative regulator; preferential binding to denatured CRP (in vitro) [54]; CR3 [55]; C3b; heparin, extracellular matrix components; microbial virulence factors; annexin-II; DNA; DNA-devoid histones [56]
C4-binding protein	Negative regulator; C4b and cofactor for the FI-mediated conversion of C4b into inactive fragments [57]
Clusterin	Negative regulator; blocking of the binding of nascent C5bC7 to cell membranes [58]; possibly inhibition of the C5bC7 complex assembly; constituent of the “sC5bC9 complex” [58], binding to sites within C7, C8, and C9 [59]
Vitronectin	Negative regulator; a constituent of the sC5bC7 complex and, to a lesser extent, inhibition of C9 polymerization [60] through the occupation of the metastable membrane-binding site of the C5bC7 complex and hindrance of its insertion into the cell membrane [61]
C1-inhibitor	Negative regulator; suicide inhibition by means of complex formation with C1r, C1s, and MASP1 and MASP2 [62,63]
Factor I	Negative regulator; C4b inactivation by conversion to inactive split products with C4BP as a cofactor [57]; proteolysis of C3b in the presence of FH [64]
CFHR1	Positive and negative regulator; extracellular matrix components; competitive inhibition of FH and LP/CP C5 convertase [65]

**Table 2:** The main functions of the complement system proteins [68,69,70].

Complement Fragments	Function
C1 complex (C1q, C1s, C1r)	pattern recognition molecule, CP- and LP-initiating protein
C1-complex and smaller subunits of C3 and C4	initiation of complement cascade through C3 and C4 activation
C3b, iC3b, C4b	opsonins
C3a, C4a, C5a	chemoattraction and leukocyte activation
C3b, C4b together with antigen–antibody complexes bound to CR2 on B lymphocytes	B-cell activation and antibody production stimulation
C3b, C4b together with antigen–antibody complexes bound to CR2 and CR3 on dendritic cells in lymphoid follicles	immune cell activation
C5b-polyC9 (MAC)	cell lysis

**Table 3:** The characteristics of receptors involved in signal transduction initiated by binding to complement fragments and other ligands [71].

Receptor	Ligands	Function
CR1	C3b/C4b AP/CP C3 convertase C5 convertase Other ligands: C1q, MBL, and iC3b/C3d(g) with low affinity	regulation of complement activation; *CR1* gene encodes for the antigens of the Knops blood group system; presentation of foreign antigens to immunocompetent cells; inhibitory B-cell receptor; soluble CR1 (sCR1) has anti-inflammatory properties
CR2	iC3b C3d(g) Other ligands: IFNa, Low-affinity IgE receptor CD23	reduction in the threshold for immune activation; regulation of complement activation; entry receptor for EBV, HIV-1; facilitation of *Cryptococcus neoformans* internalization; complement-opsonized antigen presentation
CR3	iC3b C3d(g) C3(H_2_O) Other ligands: ICAMs, Fibrinogen, Plasminogen, LPS (many others)	immune mediated adhesion and phagocytosis; outside–inside signaling; mediation of B-cell cytotoxicity towards cancer cells; “scavenger” receptor; binding of coagulation system proteins
CR4	iC3b Other ligands: ICAM-1, VCAM-1, Fibrinogen, LPS, Heparin (others)	immune mediated adhesion and phagocytosis; mediation of NK-cell complement-dependent cytotoxicity; “scavenger” receptor
CRIg	C3b iC3b	inhibition of the C3 convertase of the AP; facilitation of phagocytosis
C3aR	C3a Other ligands: C5a	binding of anaphylatoxins
C5aR1	C5a C5a-desArg Other ligands: C3a, ribosomal protein S19	intracellular signaling mechanism dependent on cell type and ligand
C5aR2	C5a, C5a-desArg	intracellular signaling (role secondary to C5aR1); internalization, retaining, and degradation of C5a; regulation of complex cellular responses.

## Data Availability

No new data were created or analyzed in this study. Data sharing is not applicable to this article.
